# Challenges for China to achieve carbon neutrality and carbon peak goals: Beijing case study

**DOI:** 10.1371/journal.pone.0258691

**Published:** 2021-11-15

**Authors:** Junfeng Hu, Jiang Wu, Chuan Zhao, Peng Wang

**Affiliations:** 1 Beijing Key Laboratory of New Energy and Low-Carbon Development, Economics and Management School, North China Electric Power University, Beijing, China; 2 Electrical and Electronic Engineering School, North China Electric Power University, Beijing, China; Central Agricultural University, INDIA

## Abstract

China has set a goal to achieve peak CO_2_ emissions before 2030 and carbon neutrality by 2060. To achieve the goals of carbon peak and carbon neutrality, China needs to address the challenge of the large and still growing CO_2_ emission base. This paper investigated the energy consumption and CO_2_ emission in Beijing from 2020–2035 based on the energy elasticity coefficient and contribution value of the sub-energy increment (CVSI) method. Beijing is one of the first cities in China to propose the "carbon peak” target as of 2020. From 2020 Beijing will strive to achieve the goal of carbon neutrality. The results show that in 2035 the CO_2_ emission in Beijing may drop to 50% of 2020. This decline would be affected by economic growth, energy efficiency and the proportion of renewable energy use. Beijing’s energy supply mainly comes from outside the region. Therefore, for Beijing, in addition to increasing the proportion of renewable energy sources outside the region, its own energy acceptance also needs to be strengthened, including strengthening energy storage construction, actively researching and promoting carbon capture and utilization of gas-fired units, which are effective ways to achieve carbon neutrality target.

## 1. Introduction

China has recently shifted from a high-speed growth stage to a high-quality growth stage, and this high-quality development will inevitably place a higher strain on the energy infrastructure and the environment. To overcome these challenges, China has set a goal to achieve peak CO_2_ emissions before 2030 and achieve carbon neutrality by 2060 [1]. It has been further announced at the Climate Ambition Summit that CO_2_ emissions, per unit of gross domestic product (GDP), will be reduced by 65% of that of 2005 levels by 2030; non-fossil energy will account for approximately 25% of primary energy consumption, forest accumulation will increase by 6 billion m^3^ in 2005, and the total installed capacity of wind power and solar power will reach 1.2 billion kWh [2].

To achieve the goals of "carbon peak" and "carbon neutrality," China needs to address the challenge of the large and still growing CO_2_ emission base. In 2019, the United States ranked the second with 4.965 billion tons of CO_2_ emissions and the European Union ranked the third with 4.111 billion tons, while China ranked first with 9.826 billion tons [3]. China’s carbon emissions have exceeded the combined emissions of the United States and the European Union, and it is expected to reach its peak in 2030. In addition, China’s energy structure makes carbon emission reduction targets more difficult to achieve than that of other countries. The world’s energy structure has experienced a transition from coal to oil to gas, and is currently undergoing a transition from oil and gas to non-fossil fuels; thus, world energy development has shifted from high- to low-carbon [4]. In 2019, coal, oil, and natural gas accounted for 57.7%, 18.9%, and 8.1% of the total energy consumption, respectively. Although coal consumption accounts for more than half of China’s total energy consumption in 2019, it is 10.8% lower than that in 2012.

With the changes in China’s industrial structure, energy consumption is rapidly transitioning to clean and low-carbon energy [5]. In 2019, natural gas, hydropower, nuclear power, wind power, and other clean energy resources accounted for 23.4% of the total energy consumption, an increase of 8.9% from 2012; non-fossil energy accounted for 15.3% of total energy consumption, an increase of 5.6% from 2012. At the same time, China’s energy utilization efficiency has significantly improved. From 2012 to 2019, energy consumption per unit of GDP has reduced by 24.4%, which is equivalent to the reduction in the energy consumption by 1.27 billion tons of standard coal. From 2012 to 2019, an average annual growth of 2.8% in energy consumption supported an average annual growth of 7% in the national economy [6].

Beijing is one of the first cities in China to propose the "carbon peak” target. The current peak of carbon emissions in Beijing has been achieved, it peaked for the first time in 2011, but returned to the peak in 2013, and then the emissions are showing a steady downward trend [7]. This study aims to predict the energy consumption in Beijing from 2020 to 2035 and provide a reference for other Chinese cities that are about to enter the peak prase.

## 2. Literature review

Studies on predicting energy consumption often use the time series and artificial neural network models. Nguyen [8] integrated the influencing factors of energy consumption of 115 economies, from 1991 to 2014, and used the dynamic panel data to estimate the determinants of energy consumption in terms of the level, intensity, and sustainability (renewable energy). Ediger and Akar [9] used the autoregressive integrated moving average (ARIMA) and seasonal ARIMA (SARIMA) methods to estimate the future primary energy demand of Turkey from 2005 to 2020. Magazzino [10] used nearly 80 years of data from Italy to investigate the relationship between energy consumption and economic growth via wavelet analysis so as to decompose the sequence into different time scales. The influence of energy consumption on economic growth can significantly be detected only at lower scales. The differences between longer scales and lower scales justify the use of wavelet approach to decompose time series into various time scales. Geem [11] used a four-variable artificial neural network model (with feedforward multilayer perceptron, error back propagation algorithm, momentum process, and scaled data structure) to effectively estimate Korea’s energy demand. Some scholars [12–15] used other methods to predict energy consumption, such as the long-term energy alternative planning (LEAP) model under five different scenarios that include Zhang’s [15] analysis benchmarks, different economic growth rates, different industrial structures, energy conservation, and integration to analyze the development trend of Beijing’s energy consumption and energy structure from 2017 to 2035.

A Sankey diagram is a tool used to visualize the flow of energy [16]. The most important feature of a Sankey diagram is that the sum of the branch widths at the beginning and the end is equal; the branch width represents the flow rate of the branch and maintains the conservation of energy. Sankey diagrams are used as visualization tools to evaluate the energy or mass balance relationship in buildings [17], cogeneration systems [18], and Heating, ventilation and air conditioning systems(HVAC) [19], as well as in regional energy flow and greenhouse gas emissions. Donglin et al. [20] analyzed the energy supply, intermediate conversion, and terminal consumption in Shandong Province in 2017 by drawing an energy flow diagram and compared the energy consumption with that in 2008 based on this analysis so as to offer policy recommendations. Wang et al. [21] used a Sankey diagram to obtain China’s energy flow map, which visually expressed China’s energy structure and CO_2_ emissions, and provided a reference for policy recommendations.

Economic growth and changes in the industrial structure have a significant impact on energy consumption. However, most domestic energy consumption forecasts are based on time series analysis and grey forecasting that use single or combined models for total forecasting or forecasting a certain energy type. There are few studies that used energy flow diagrams to calculate regional energy consumption levels [22]. Starting from terminal industries’ energy consumption, total energy demand is calculated via reverse analysis.

The role of renewable energy consumption has been investigated as it plays a prominent role in carbon emission and carbon emission also influence renewable energy consumption [23]. In the long run, carbon emissions are positively correlated with energy consumption, and energy consumption has a limited short-term impact on carbon emissions [24].The energy balance sheet provides sufficient data support for energy-related carbon emissions accounting. This article uses the calculation method provided in the "Requirements for carbon dioxide emission accounting and reporting" [25] for carbon emissions accounting which is designed by Beijing. However, there is relatively little literature on the challenges China will encounter in achieving its carbon neutrality and carbon peak goals.

## 3. Research methods

To predict the total energy consumption in Beijing, the following research methods were used in this study.

Based on the policy requirements and real world conditions, the future GDP growth rate of Beijing was predicted. After the overall GDP growth rate was obtained, the GDP growth rate *β*_i_ was calculated by estimating the contribution of three industries (Primary Industry, Secondary Industry, Tertiary Industry) using the moving average method.

Based on the historical data, the elasticity coefficient of energy consumption, *e*_*i*_, was predicted. Using Eq (1), the growth rate of energy consumption by each industry was obtained. Then, the energy consumption increments of the terminal industries, Δ*E*_*i*_, were calculated.


$$e_{i}=\frac{\alpha_{i}}{\beta_{i}}.$$ (1)

Then, the contribution value of the sub-energy increment (CVSI) [26] was used to predict different types of energy used by the industries. CVSI refers to the contribution of each energy demand increment to the energy consumption elasticity coefficient, i.e., the product of the proportion of each energy demand increment to the total energy consumption increment in the current year and the energy consumption elasticity coefficient. The sum of the CVSIs is the energy consumption elasticity coefficient (Eq (2)).

$$C_{ij}=\frac{\Delta E_{ij}}{{\Delta E}_{i}}e_{i},$$ (2)

where *C*_*ij*_ is the CVSI of energy type j in industry i,

Δ*E*_*i*_ is the energy consumption increment of industry i,

Δ*E*_*ij*_ is the increment of energy type j in industry i.

Δ*E*_*ij*_ reveals the energy consumption by different industries in the forecast period.

Finally, using the energy conversion structure in the Sankey diagram, the total energy consumption of Beijing was determined.

Sankey diagram was used to understand the energy supply, intermediate conversion, and terminal consumption patterns in Beijing. The data used in this study were obtained from the 2020 Beijing Statistical Yearbook(http://nj.tjj.beijing.gov.cn/nj/main/2020-tjnj/zk/indexch.htm). To prevent the energy flow diagram from being too complicated, this study combines some energy categories and terminal industries based on related literature [27] and makes some improvements. (Tables 1 and 2). As shown in Eq (3), *K*_*ij*_, *D*_*ij*_, and *S*_*ij*_ are the initial demand, end demand, and conversion volume of the j-th energy type consumed by the i-th industry, respectively.


$$K_{ij}=\Sigma D_{ij}+\Sigma S_{ij}.$$ (3)

**Table 1:** Consolidated results of energy categories.

Original statistical entry	Merged entry
Raw coal, washed clean coal, other washed coal, coal products, coke	Coal
Base oil, gasoline, kerosene, diesel, fuel oil, naphtha, lubricating oil, paraffin, solvent oil, petroleum pitch, petroleum coke, liquefied petroleum gas, refinery dry gas, other petroleum products	Oil
Natural gas, liquefied natural gas	Gas
Heat	Heat
Electricity	Electricity
Other energy	Other energy

**Table 2:** Consolidated results of industry categories.

Original statistical entry	Merged entry
Agriculture, forestry, animal husbandry, and fishery	Primary industry
Mining industry, manufacturing industry, electricity, gas and water production and supply industry, construction industry	Secondary industry
Wholesale and retail industry, accommodation and catering industry, information transmission, software and information technology service industry, financial industry, real estate industry, leasing and business service industry, scientific research and technology service industry, water conservancy, environment and public facilities management industry, residents services, repair and other service industries, education, health and social work, culture, sports and entertainment industries, public administration, social security and social organizations	Other tertiary industry
Transportation, storage, and postal transportation	Transportation
Household consumption	Household consumption

Based on this analysis, energy consumption of the terminal industry in the energy flow chart was obtained, and a more detailed division was made. This study adopted the elastic coefficient method and the CVSI method to predict the energy consumption level of Beijing’s terminal sub-sectors in 2025, 2030, and 2035, and then inversely inferred the initial and final energy demand based on the energy conversion structure in the energy flow chart of Beijing in 2019.


$$CO_{2}=\sum {NCV}_{i}*{FC}_{i}*{CC}_{i}*{OF}_{i}*44/12$$ (4)

Where *CO*_2_ is the total amount of carbon dioxide emitted due to the consumption of various fossil fuels (for coal, oil, and natural gas); *NCV*_*i*_ is the average low calorific value of the i-th fossil fuel; *FC*_*i*_ is the consumption of the i-th fossil fuel; *CC*_*i*_ is the carbon content per unit calorific value of the i-th fossil fuel, in units of ton of carbon per GJ; *OF*_*i*_ is the carbon oxidation rate of the i-th fossil fuel.

## 4. Results

### 4.1. Energy flow chart of Beijing

**Fig 1:**
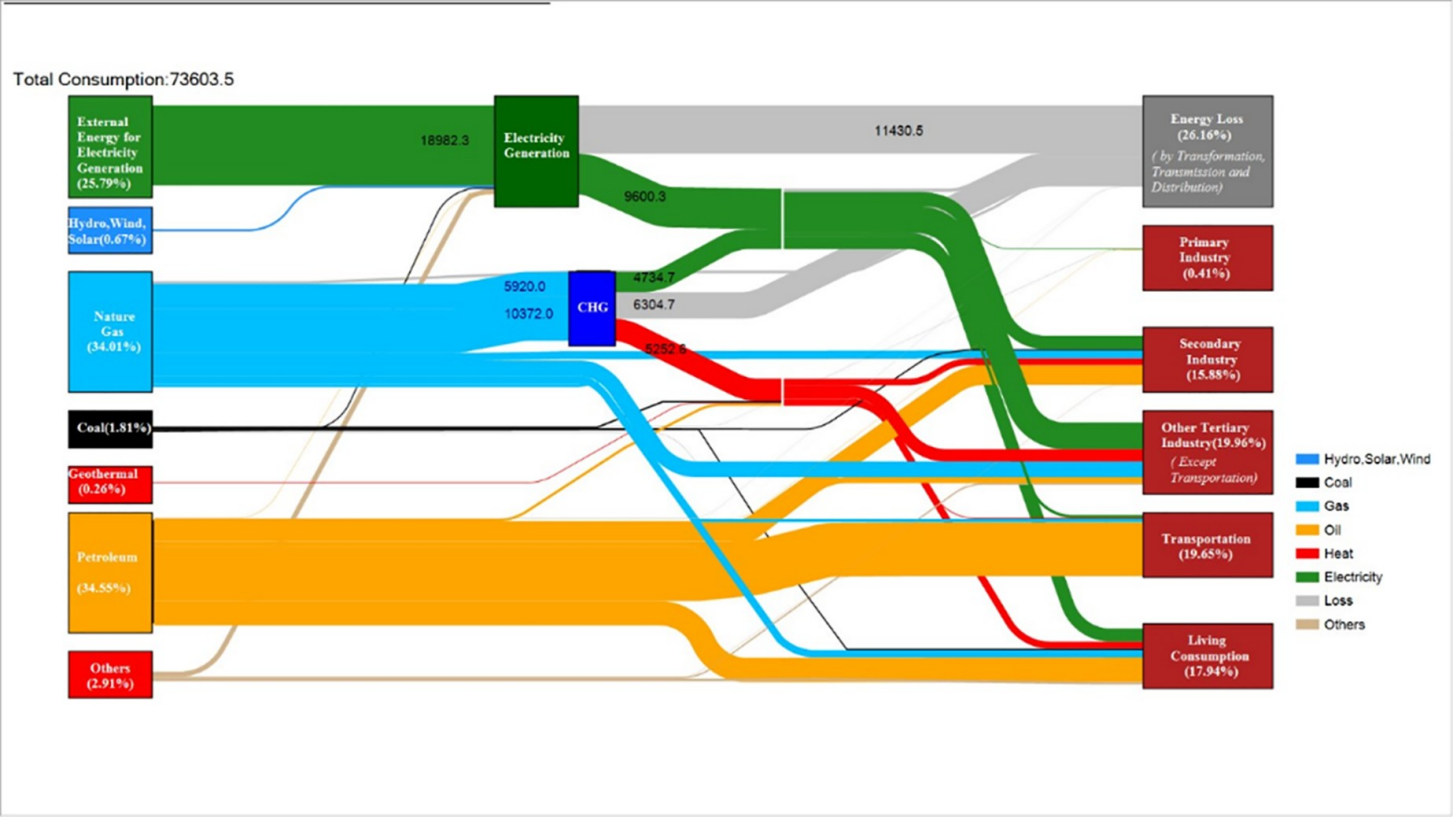
Energy flow diagram of Beijing in 2019 (obtained by the electrothermal equivalent method).

In this study, energy consumption in Beijing was analyzed by using the relevant data from the "Beijing Statistical Yearbook 2020”. Thousand tons of coal equivalent (ktce) was used as the energy unit. The energy flow map of Beijing in 2019 is displayed in Fig 1.

The calculation of coal consumption for power generation refers to the conversion of electric power into standard coal according to the average coal consumption for thermal power generation in that year and is more commonly used in China, whereas the calculation of electric heating equivalent refers to the conversion of electric power into standard coal according to the thermal work equivalent and is generally used for international energy data. The standard conversion factor is 10,000 kwh = 1.229 ttce. Since the data of the electrothermal equivalent method are not available in the "Beijing Statistical Yearbook 2020," we have made some modifications to the data.

Because the energy efficiency of the terminal sector is not considered in this study, the energy flow diagram calculated using the electrothermal equivalent method does not involve the loss of the terminal sector; only the loss of conversion, transmission, and distribution is considered. To make the calculation more convenient, a coal consumption method for power generation was adopted to obtain the start- and end-point energy consumption and energy conversion.

Compared with the energy flow diagrams in 2013–2018, the energy consumption structure of Beijing shows that the proportion of coal consumption is decreasing and that of natural gas is increasing every year. The proportion of coal has dropped from 23.3% in 2013 to 1.8% in 2019, and the proportion of natural gas, oil, and electricity has increased from 18.1% to 34.0%, from 32.2% to 34.6%, and from 25.3% to 26.5%, respectively. According to the energy flow diagram, the transmission and distribution loss rate is 6.3%, the gas conversion loss rate is 5.8%, the ratio of gas conversion to electricity is 63.7%, and the ratio of gas conversion to heat is 36.3%.

### 4.2. Energy consumption in Beijing

This study adopts the elastic coefficient method to analyze and forecast Beijing’s energy consumption. The method to predict the energy consumption elasticity coefficient comes from the concept of "elasticity" in economics, which expresses the degree of sensitivity to changes between two variables. The energy consumption elasticity coefficient is a technical and economic indicator that reflects the relationship between energy and national economic development. It refers to the ratio of the average growth rate of energy consumption in a certain period to the average growth rate of GDP or the industrial and agricultural production in the same period.

A relatively large energy consumption elasticity coefficient indicates that the economic development is heavily dependent on energy whereas a relatively small energy consumption elasticity coefficient indicates that economic development is less dependent on energy. A negative value indicates economic growth while energy consumption decreases. It occurs when the consumption coefficient (the amount of energy consumed per unit of GDP) decreases faster than the economic growth rate.

#### 4.2.1. First stage in Beijing energy consumption (2000–2011)

**Fig 2:**
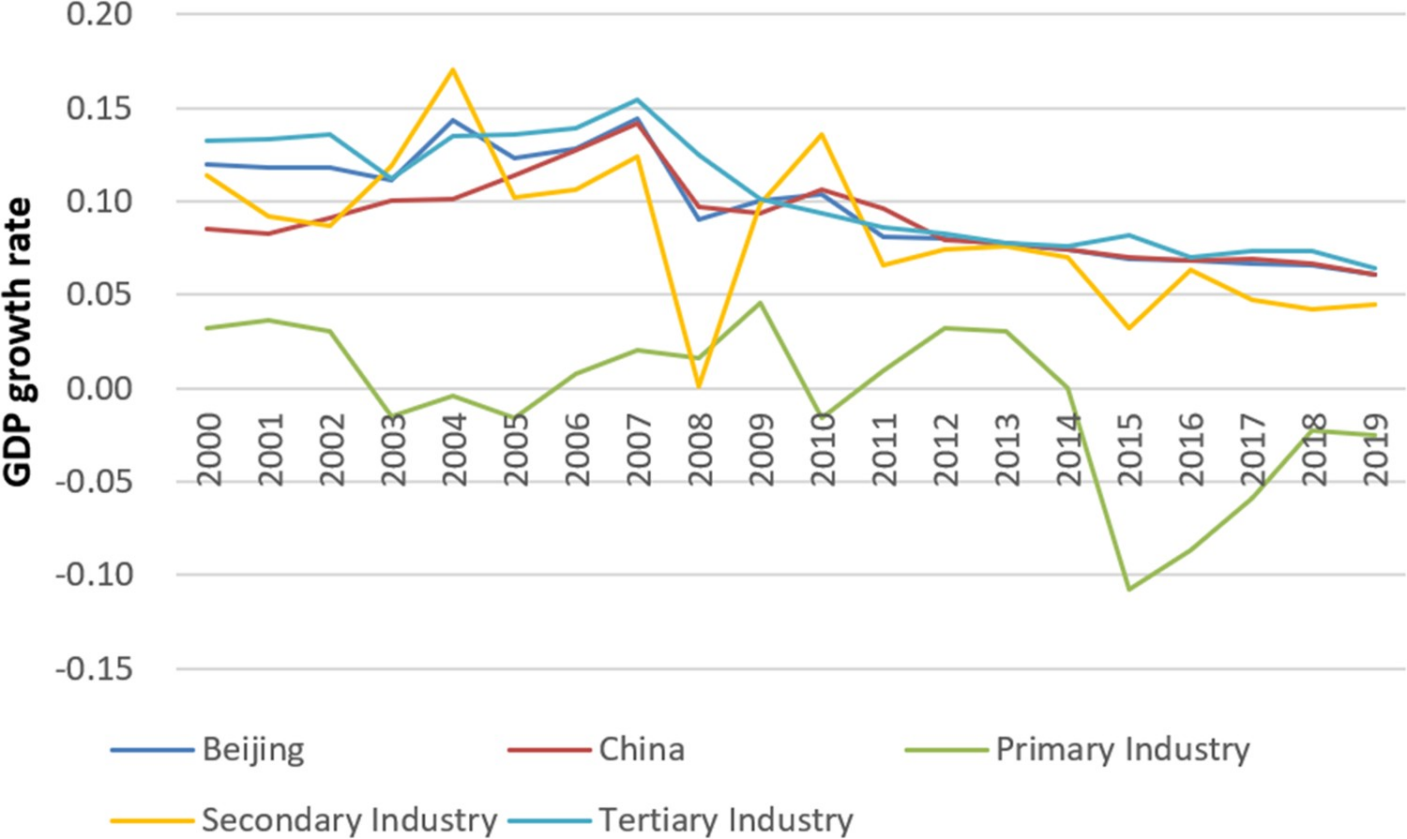
GDP growth of various industries in Beijing.

**Fig 3:**
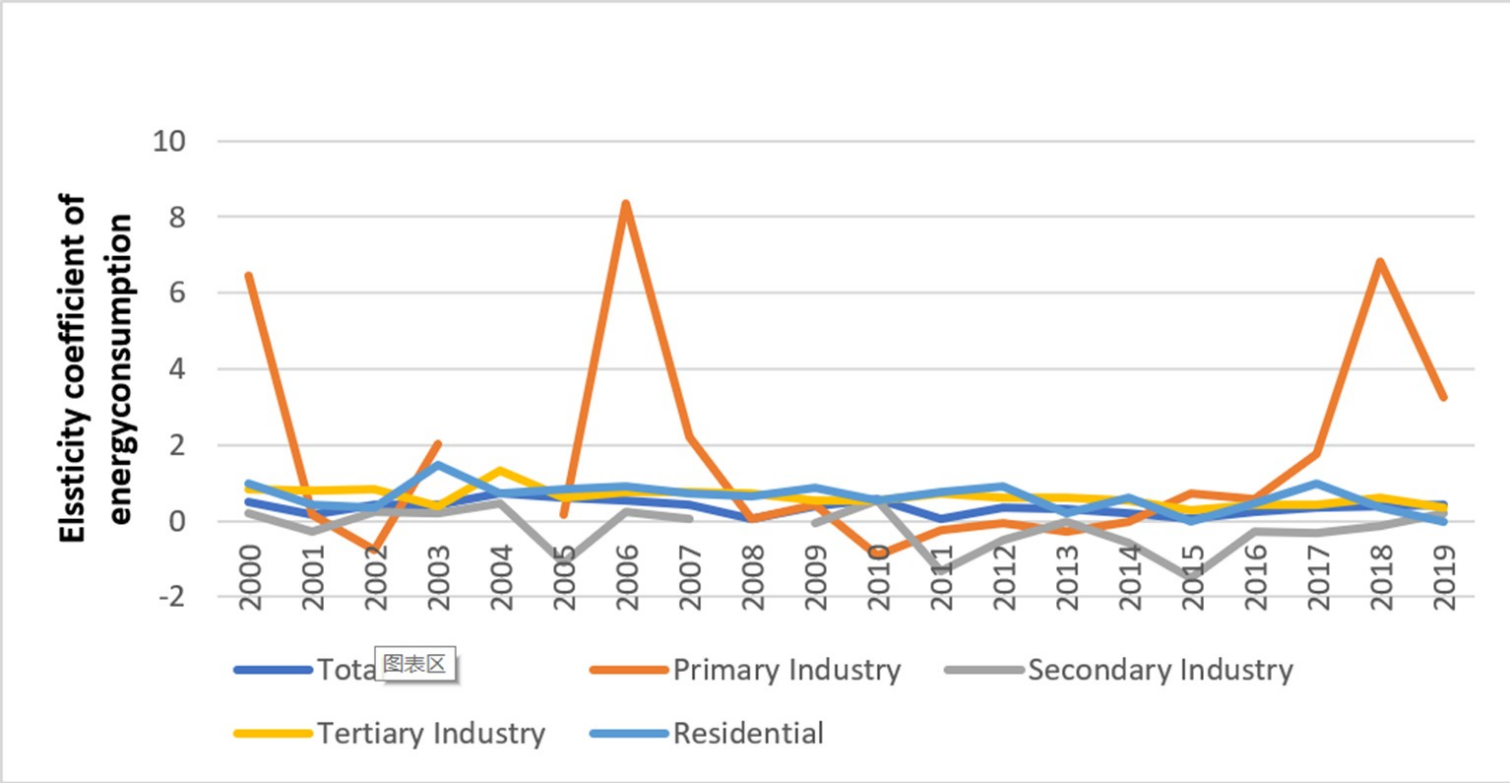
Elasticity coefficient of energy consumption in Beijing from 2000 to 2019.

The economy grew rapidly from 2000 to 2011. At this stage, the annual growth rate of Beijing’s GDP remained above 8%, and the highest rate was 14.4% in 2007. The annual growth rate was much higher than the global economic growth rate (Fig 2). Fig 3 shows the calculated elasticity coefficient of energy consumption by industry from 2000 to 2011.

The energy consumption elasticity coefficient at this stage fluctuates greatly, except for a few years; the overall value is above 0.40, and the average value is 0.42. The energy elasticity coefficient of the primary industry also fluctuates greatly but with a downward trend. In 2000, 2004, and 2006, the values are relatively large, but the average value for the remaining years is 0.35. Overall, the secondary industry shows a downward trend. The 2008 value is considered an abnormal point, and the average value for the remaining years is -0.1. The tertiary industry and residential consumption values fluctuate with the average values of 0.74 and 0.78, respectively.

#### 4.2.2. Second stage in Beijing energy consumption (2012–2019)

From 2012 to 2019, following the economic crisis, China’s economy entered a period of adjustment and transformation and gradually arrived at a new normal of development. The GDP growth rate decreased from 8.0% in 2012 to 6.1% in 2019 (Fig 2). Economic development has shifted its focus more toward quality and benefits. Fig 3 shows the elasticity coefficient of energy consumption by industry from 2012 to 2019.

In general, the energy consumption elasticity coefficient at this stage is below 0.4, with an average value of 0.26. The average energy consumption elasticity coefficient of the primary industry is 1.83, the average of the secondary industry is -0.38, the tertiary industry has a gradual downward trend with an average of 0.49, and living consumption has an average value of 0.44.

#### 4.2.3. Third stage in Beijing energy consumption (2020–2035)

From 2020 to 2035, the global economic growth will continue to slow down, and China will be in a critical period of transforming its development mode, optimizing economic structure, and transforming its growth momentum. Beijing will also face many problems and challenges in achieving urban renewal and high-quality development. The 18th National Congress of the Communist Party of China recommended accelerating the improvement of the socialist market economy system and the transformation of economic development methods, and shifting the focus toward improving quality and efficiency. The report of the 19th National Congress of the Communist Party of China suggests that “building a modern economic system” must insist on quality and efficiency first. China’s economic growth will enter a stage of high-quality and high-efficiency growth period.

The 2018 Beijing Municipal Government’s work report set the regional GDP growth target at 6.5%, and the 2019 and 2020 reports set this target at 6.0–6.5% and 6.0%, respectively. This growth rate is in line with China’s GDP growth rate. The World Bank, China Petroleum Institute of Economics and Technology, and other institutions and experts predict that China’s GDP growth rate will be approximately 6% in the next 10 years.

The comparison of Beijing’s GDP growth rate from 2000 to 2019 and China’s GDP growth rate in Fig 2 show the convergence of both rates since 2012. With 2019 as the base year, to estimate the future growth, the GDP growth rate during the forecast period was divided into two scenarios:

**High growth rate scenario:** 10% GDP growth in 2018–2025, 9% in 2026–2030 and 8% in 2031–2035.

Mid growth rate scenario: 6.0% in 2018–2030 and 5.5% GDP growth in 2031–2035.

**Low growth scenario:** 3% in 2018–2030 and 2.5% GDP growth in 2031–2035.

Energy consumption elasticity coefficient is also divided into two scenarios:

**High energy efficiency scenario:** The elasticity of energy consumption in various industries is at a relatively low level.

**Low energy efficiency scenario:** The elasticity coefficient of energy consumption in various industries is at a relatively high level.

The industry contribution rate refers to the ratio of the incremental value of each industry to the incremental value of regional GDP. Since the primary industry originally accounts for a small proportion of GDP and has a trend ranging from negative growth to zero growth (Fig 2), the primary industry’s GDP remains at the 2019 level. Using the moving average method and taking n = 10, the average contribution rates of the secondary and tertiary industries from 2020 to 2035 are 17% and 83%, respectively. Using these values, the GDP growth of each industry during the forecast period and for the period 2020–2035 was calculated (Table 3).

**Table 3:** GDP growth rate of various industries and stages.

Average GDP growth rate (high growth rate scenario)	2000–2011	2012–2019	2020–2025	2026–2030	2031–2035
Beijing	12%	7%	10%	9%	8%
Primary Industry	1%	-3%	0	0	0
Secondary Industry	10%	6%	10.50%	10.41%	10.18%
Tertiary Industry	12%	8%	9.93%	9.94%	9.79%
Average GDP growth rate (mid growth rate scenario)	2000–2011	2012–2019	2020–2025	2026–2030	2031–2035
Beijing	12%	7%	6%	6%	5.5%
Primary Industry	1%	-3%	0	0	0
Secondary Industry	10%	6%	5.40%	5.33%	5.02%
Tertiary Industry	12%	8%	6.16%	6.16%	5.62%
Average GDP growth rate (low growth rate scenario)	2000–2011	2012–2019	2020–2025	2026–2030	2031–2035
Beijing	12%	7%	3%	3%	2.5%
Primary Industry	1%	-3%	0	0	0
Secondary Industry	10%	6%	3.17%	3.16%	2.99%
Tertiary Industry	12%	8%	2.97%	2.97%	2.82%

Using the historical data from the first and second stages and the moving average method (n = 10), the elasticity coefficient of energy consumption during the forecast period was calculated (Table 4).

**Table 4:** Elasticity coefficient of energy consumption in different industries and stages.

Elasticity coefficient of energy consumption	2020–2025	2026–2030	2031–2035
Primary Industry	1.81	2.32	2.20
Secondary Industry	-0.41	-0.31	-0.35
Tertiary Industry	0.49	0.48	0.48
Household consumption	0.44	0.42	0.42

### 4.3. Final energy consumption forecasting

#### 4.3.1. Total energy consumption forecasting

**Fig 4:**
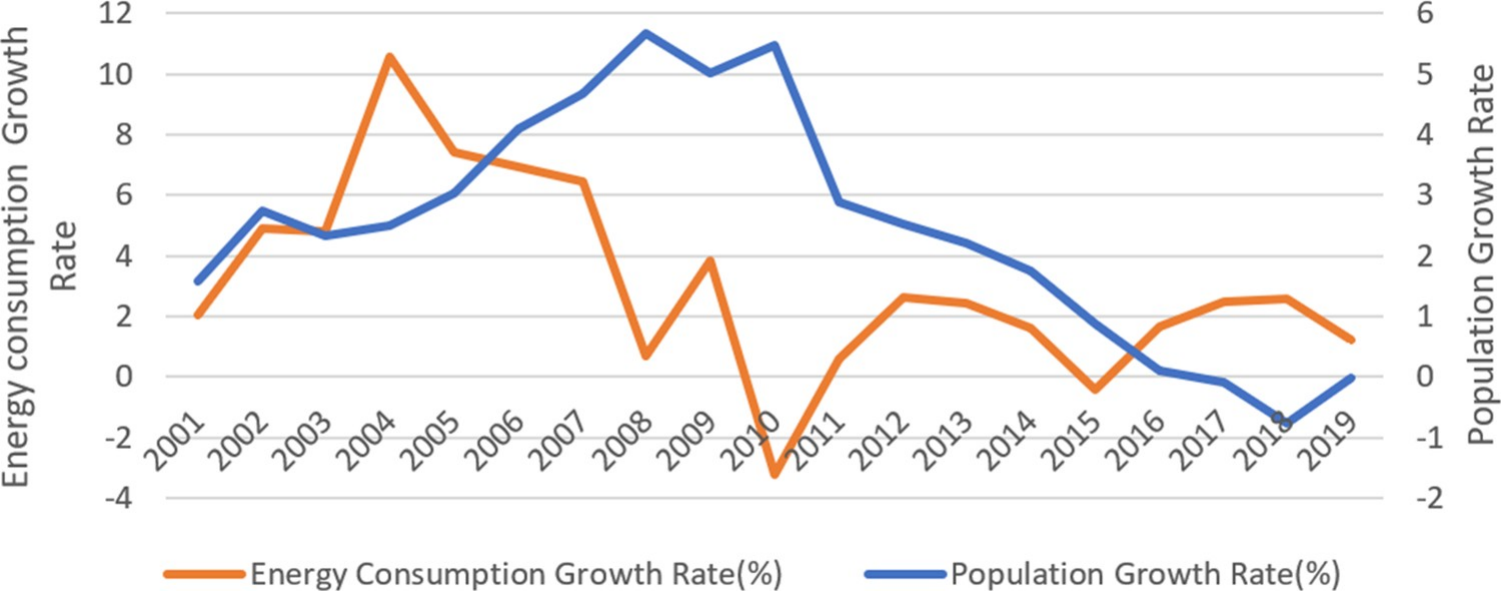
Energy consumption and population of Beijing.

In addition, we take the population growth rate into account in energy consumption forecasts because China’s population growth is about to reach an inflection point and would have a great impact on energy consumption. The population growth rate of Beijing in the past two decades is shown in the Fig 4. The growth rate of population shares the same trend with energy consumption, except for few years. According to the predictions and plans of the Chinese government, the population of China will reach its peak in 2030. Department of Economic and Social Affairs of the United Nations forecasts the population of China in various scenario. Based on the above situation, we use the results in Constant-fertility which assume the growth rate of population will drop to 2.5‰ in the first period (2020–2025), 0.07‰ in second period (2026–2030), and -0.07‰ in third period (2031–2035).

In order to add change of population to energy forecast model, we use the linear regression of population growth rate(Rp) to Elasticity coefficient of energy consumption(EC). For data on population growth rate, see Beijing Statistical Yearbook. However, the selected variables are time series, to avoid spurious regression, the series need to be taken a unit root test. The results of unit root test are shown in Table 5, which suggest EC is stationary and Rp is first-order difference stationary.

**Table 5:** Augmented Dickey-Fuller unit root test.

	ADF	
	t-Statistic	prob
Ec	-3.86	0.0370
Rp	-0.69	0.4000
dRp	-3.75	0.0009

Based on the results of the unit root test, equation form is set up as follow. To estimate the coefficients of the equation, we use the fully modified least squares method. The regresssion results are shown as following.


$$Ec=13.04*{dR}_{p}+0.37$$ (5)

t=(2.19)(7.18)

$$R^{2}=0.17\;D.W=2.16\;F=3.72$$

We can get the energy consumption in the forecast period considering the effect of population growth. Using the difference between Elasticity coefficient of energy consumption in the forecast period and in 2019, we obtain a correction value that takes the population change into account. The results are shown in Table 6.

**Table 6:** Population growth rate correction.

Years	Average annual rate of population change (‰)	Elasticity coefficient of energy consumption	Difference
2012–2019	8.1	0.4815	-
2020–2025	2.5	0.4110	-0.0705
2026–2030	0.7	0.3513	-0.1303
2030–2035	-0.7	0.35654	-0.1250

Based on the analysis of the energy elasticity coefficient, the energy elasticity coefficient interval was determined; the secondary, tertiary, and household consumption intervals were set to [-0.32,-0.22], [0.47,0.63], and [0.42,0.61], respectively; and the total energy consumption by industry during the forecast period was calculated (Table 7).

**Table 7:** Forecast value of terminal energy consumption by industry based on elasticity coefficient method (ktce).

		Scenario 1			Scenario 2			Scenario 3		
Year	2019	2025	2030	2035	2025	2030	2035	2025	2030	2035
Primary Industry	558	558	558	558	558	558	558	558	558	558
Secondary Industry	18,502	1,498	1,308	1,148	1,548	1,386	1,246	1,662	1,546	1,442
Tertiary Industry	37,573	4,951	6,332	7,982	5,240	7,010	9,225	4,352	5,111	5,910
Household consumption	16,914	2,166	2,676	3,224	2,333	3,048	3,860	1,921	2,214	2,519
Sum	73,547	9,173	10,874	12,912	9,679	12,002	14,889	8,493	9,429	10,429
		Scenario 4			Scenario 5			Scenario 6		
Year		2025	2030	2035	2025	2030	2035	2025	2030	2035
Primary Industry		558	558	558	558	558	558	558	558	558
Secondary Industry		1,689	1,593	1,505	1,738	1,610	1,498	1,754	1,694	1,638
Tertiary Industry		4,629	5,600	6,655	4,089	4,342	4,597	4,162	4,566	4,983
Household consumption		2,056	2,460	2,898	1,824	1,923	2,011	1,866	2,043	2,203
Sum		8,932	10,211	11,616	8,208	8,433	8,664	8,340	8,862	9,381

Scenario 1: High growth and high energy efficiency scenario.

Scenario 2: High growth and low energy efficiency scenario.

Scenario 3: Mid growth and high energy efficiency scenario.

Scenario 4: Mid growth and low energy efficiency scenario.

Scenario 5: Low growth and high energy efficiency scenario.

Scenario 6: Low growth and low energy efficiency scenario.

#### 4.3.2. Forecasting energy consumption structure of different end users

The energy consumption elasticity coefficient method is relatively reliable for predicting the total energy consumption, but when applied to the analysis of energy consumption forecasting, the regularity is not obvious.

The contribution of each energy increment by energy type and sub-industry from 2014 to 2019 calculated using Eq (2) is shown in Table 8.

**Table 8:** CVSI by energy sector from 2014 to 2019.

		Coal	Oil	Gas	Heat	Electricity	Others
2014	Secondary Industry	-0.29	-0.12	-0.02	-0.01	-0.12	0.00
	Tertiary Industry	0.02	0.27	0.04	0.13	0.06	0.01
	Household consumption	0.03	0.16	0.07	0.09	0.24	0.03
2015	Secondary Industry	-0.96	0.21	0.42	-0.06	-1.11	0.00
	Tertiary Industry	-0.11	0.15	0.11	0.01	0.11	0.02
	Household consumption	-0.10	0.34	0.13	0.07	0.02	0.00
2016	Secondary Industry	-0.10	-0.25	0.03	0.00	0.05	0.00
	Tertiary Industry	-0.17	0.26	0.03	-0.06	0.38	0.00
	Household consumption	-0.19	0.13	-0.10	0.08	0.49	0.00
2017	Secondary Industry	-0.84	0.49	0.38	0.05	-0.36	-0.02
	Tertiary Industry	-0.20	0.33	0.02	0.03	0.25	-0.02
	Household consumption	-0.52	0.19	0.54	0.05	0.56	0.13
2018	Secondary Industry	-3.88	-3.96	6.64	5.95	-5.76	0.89
	Tertiary Industry	-0.16	0.14	0.21	0.15	0.29	0.01
	Household consumption	8.92	-2.83	3.24	-0.47	-6.63	-1.87
2019	Secondary Industry	0.00	0.12	-0.01	-0.01	0.10	0.00
	Tertiary Industry	-0.01	0.04	0.03	-0.03	0.29	0.02
	Household consumption	-0.19	0.11	0.10	0.04	-0.13	0.06

The energy structure of Beijing has changed in recent years; coal consumption has gradually decreased. In contrast, the proportion of natural gas and electricity has gradually increased, and oil consumption has stabilized. The proportion of energy consumption by the primary industry is relatively small, and the main energy type is electricity with a relatively fixed value. Therefore, the terminal energy consumption by the primary industry remains unchanged, and it is in the form of electricity, according to the predicted value in Table 7.

The moving average method was used to determine the contribution of each industry by energy type.

Prediction of contribution of non-coal energy sources for the secondary industry

In recent years, the energy consumption of secondary industries in Beijing has gradually decreased. The incremental contributions of oil, natural gas, heat, and electricity were calculated using the moving average method as 0.15, 0.24, 0.01, and -0.36, respectively.

Prediction of contribution of non-coal energy sources for the tertiary industry

In recent years, the energy consumption of tertiary industries in Beijing has gradually increased. The incremental contributions of oil, natural gas, heat, and electricity were calculated using the moving average method as 0.26, 0.05, 0.01, and 0.24, respectively.

Prediction of contribution of non-coal energy sources for household consumption

In recent years, the domestic energy consumption of Beijing has also gradually increased. The incremental contributions of oil, natural gas, heat, and electricity were calculated using the moving average method as 0.20, 0.22, 0.07, and 0.40, respectively.

We also considered the development trends of other energy sources and predicted the incremental contribution of coal. Using this value, we predicted the incremental contribution of each energy type by industry in the forecast period under different scenarios.

CVSI of coal = energy consumption elasticity coefficient—CVSI of other energy types.

#### 4.3.3. Forecasting energy consumption by industry

The predicted values of oil, gas, heat, and electricity were obtained using the predicted value of the terminal energy consumption. Because the predicted value of coal in some years is negative, it is allocated to oil, gas, heat, and electricity in proportion to obtain the values in Table 9.

**Table 9:** Forecast values of terminal energy consumption by energy and industry (ktce).

Scenario		Industries	Coal	Oil	Gas	Heat	Electricity	Sum
Scenario 1	2025	Secondary Industry	0	607.67	424.51	157.83	308.30	1,498.31
		Tertiary Industry	0	1995.97	552.09	332.60	2070.51	4,951.17
		Household consumption	0	669.17	351.48	206.01	939.00	2,165.66
	2030	Secondary Industry	0	603.16	494.85	140.92	68.97	1,307.89
		Tertiary Industry	0	2634.30	676.33	359.42	2661.76	6,331.80
		Household consumption	0	769.21	492.32	243.28	1171.06	2,675.87
	2035	Secondary Industry	0	577.23	516.19	125.73	71.44	1,147.71
		Tertiary Industry	0	3395.58	825.45	392.84	3368.18	7,982.06
		Household consumption	0	884.74	635.74	284.91	1419.09	3,224.49
Scenario 2	2025	Secondary Industry	0	629.60	441.68	163.13	313.45	16,779.40
		Tertiary Industry	0	2114.73	583.53	350.26	2191.81	45,888.00
		Household consumption	0	718.38	381.10	221.44	1011.98	20,196.30
	2030	Secondary Industry	0	644.07	532.76	149.54	59.62	15,480.10
		Tertiary Industry	0	2923.99	746.16	392.03	2948.31	54,206.30
		Household consumption	0	869.52	567.42	275.80	1335.09	23,413.10
	2035	Secondary Industry	0	634.46	573.80	136.82	99.26	14,349.10
		Tertiary Industry	0	3939.14	948.67	442.16	3895.26	63,118.00
		Household consumption	0	1047.93	772.50	338.90	1701.08	26,814.40
Scenario 3	2025	Secondary Industry	0	590.05	327.40	171.41	572.87	1661.73
		Tertiary Industry	0	1721.29	497.29	319.05	1814.30	4351.94
		Household consumption	0	623.43	281.79	188.58	827.40	1921.20
	2030	Secondary Industry	0	588.32	372.03	161.15	424.13	1545.64
		Tertiary Industry	0	2087.98	559.78	320.93	2141.89	5110.58
		Household consumption	0	662.31	381.46	206.36	964.25	2214.38
	2035	Secondary Industry	0	581.29	402.55	151.73	306.30	1441.88
		Tertiary Industry	0	2469.63	627.45	326.91	2486.43	5910.42
		Household consumption	0	714.26	473.47	227.10	1104.35	2519.18
Scenario 4	2025	Secondary Industry	0	598.15	329.92	174.19	587.07	1689.33
		Tertiary Industry	0	1840.74	525.37	331.40	1931.48	4628.99
		Household consumption	0	658.83	309.76	200.16	886.81	2055.57
	2030	Secondary Industry	0	602.68	377.30	165.90	446.78	1592.67
		Tertiary Industry	0	2297.16	610.03	344.23	2348.58	5600.00
		Household consumption	0	730.09	429.71	228.15	1072.49	2460.44
	2035	Secondary Industry	0	602.27	412.64	158.16	331.67	1504.75
		Tertiary Industry	0	2791.15	702.75	359.77	2801.48	6655.15
		Household consumption	0	815.79	550.59	260.10	1271.52	2898.00
Scenario 5	2025	Secondary Industry	0	587.60	292.06	177.98	680.08	1737.72
		Tertiary Industry	0	1592.60	476.28	319.78	1700.57	4089.22
		Household consumption	0	615.10	243.93	183.56	780.95	1823.54
	2030	Secondary Industry	0	590.09	348.66	166.88	504.40	1610.02
		Tertiary Industry	0	1716.57	496.51	319.06	1810.11	4342.25
		Household consumption	0	623.63	282.54	188.69	828.36	1923.21
	2035	Secondary Industry	0	585.72	387.17	156.84	368.16	1497.89
		Tertiary Industry	0	1840.57	517.25	319.10	1920.36	4597.29
		Household consumption	0	633.54	314.05	193.67	869.57	2010.83
Scenario 6	2025	Secondary Industry	0	592.08	292.85	179.65	689.91	1754.49
		Tertiary Industry	0	1620.01	485.00	326.06	1730.54	4161.62
		Household consumption	0	630.77	248.29	188.10	798.93	1866.09
	2030	Secondary Industry	0	597.76	327.19	174.62	594.83	1694.40
		Tertiary Industry	0	1811.01	519.90	330.64	1904.38	4565.94
		Household consumption	0	656.87	305.90	199.35	881.13	2043.26
	2035	Secondary Industry	0	601.21	356.12	169.83	511.02	1638.17
		Tertiary Industry	0	2007.32	556.12	335.85	2083.50	4982.78
		Household consumption	0	683.36	354.66	210.06	954.47	2202.54

## 5. Discussion

According to the energy flow diagram of Beijing in 2019, the initial energy supply value can be predicted to determine the level of energy supply in Beijing that should be available.

The conversion of oil products is ignored because of the very small quantity; only natural gas is converted into electricity and heat. The amount of natural gas used for thermoelectric conversion is reversed through terminal heat consumption. According to the 2019 energy flow diagram, the ratio of gas-to-heat conversion to electricity-to-heat conversion was used to obtain the total amount of natural gas used for energy conversion and the amount of converted electricity.

**Fig 5:**
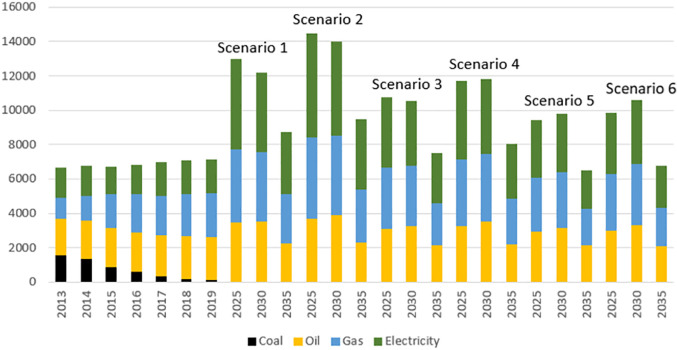
Forecast scenarios of energy consumption in Beijing.

According to the gas conversion loss rate in the energy flow diagram, the amount of gas used for energy conversion was calculated as loss, ignoring the conversion of oil products and assuming that energy consumption of the primary industry is included in the transferred electricity while considering the transmission loss, the total amount of energy consumption in Beijing was predicted. With the changes in Beijing’s industrial structure and low-carbon environmental protection requirements, the renewable energy demand will increase in the future, and the proportion of natural gas will increase although, its growth rate will gradually decline (Fig 5).

**Fig 6:**
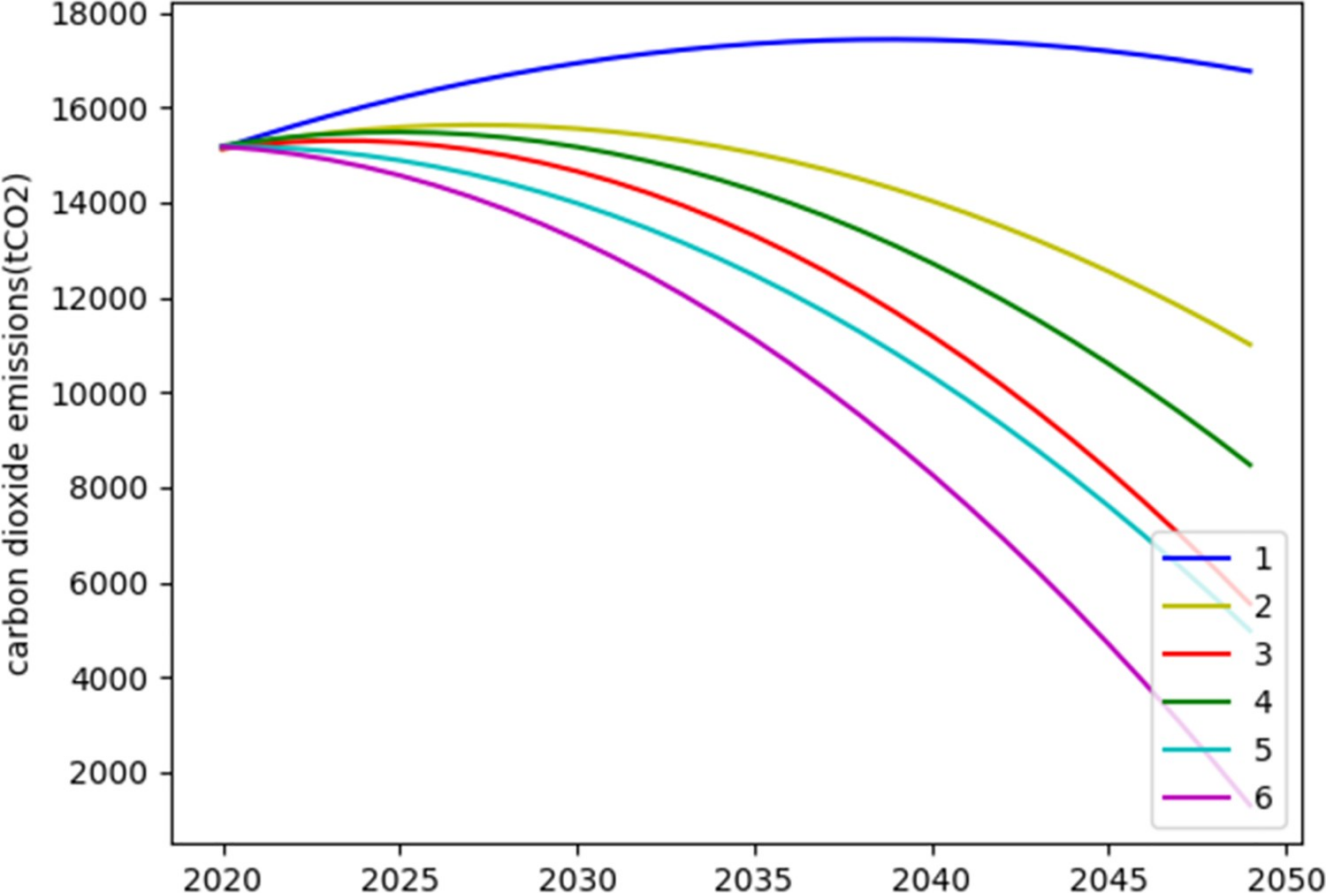
Carbon dioxide emissions in mid growth scenario and low growth scenario.

We choose the two most probable economic growth scenarios-medium growth and low growth scenarios, and both scenarios are assumed to be in high energy efficiency, because we want to Fig out how much effort it will take to ensure that carbon dioxide emissions are always reduced and achieve carbon neutrality by 2050 under an optimistic scenario. According to the energy consumption data of the above scenarios, combined with the Sankey diagrams, we can get the carbon emission data for 2025–2035, and the carbon emission data for 2035–2050 under the assumption of the same trend as shown in Fig 6.

In Fig 6, secnario 1–3 show the mid growth sceniario and 4–6 show the low growth sceniario. Different scenarios represent different share of renewable energy, renewable energy heating and transportation electrification settings. The results show that in the mid growth scenario, Beijing must promote measures such as the share of renewable energy, transportation electrification and the proportion of renewable energy heating, which should reach 15%, 20%, 25%; 30%, 50%, 70%; and 21%, 34%, 47% respectively between 2025 and 2035. In the low growth scenario, when the share of renewable energy reaches 25%, 42%, and 59% between 2025–2035, the carbon neutral goal is likely to be achieved by 2050 under the same trend.

## 6. Policy implications

In this study, Beijing’s economic development was divided into three stages, and the future GDP growth rate of Beijing’s sub-industries was predicted. The energy elasticity coefficient and CVSI method was used to obtain the final energy consumption and its structure of Beijing. Considering the share of renewable energy based on the energy structure, this article finally obtains the CO_2_ emission status of Beijing from 2020 to 2035.

Based on the results of this study, the following policy recommendations are proposed:

(1) According to the forecasting results, natural gas will have a relatively high growth rate in the future. In the next 15 years, the growth rate of externally adjusted power (including local primary power production) will increase each year, and Beijing’s energy structure will gradually shift to clean energy. To meet the demand for natural gas and electricity, Beijing should speed up the construction of gas and electricity distribution channels, adjust the local energy distribution in a timely manner, increase local primary power production, and make structural adjustments while meeting the increased demand.

(2) China’s energy intensity in 2019 was 1.3 times the world average, which is much higher than that of developed countries such as the United States, European countries, and Japan. This also shows that China has great potential for energy conservation and efficiency improvement. China should take significant measures to transition to a high-efficiency energy system as soon as possible.

(3) China needs to transform its economic development mode from a speed type to a quality type. Speed-based economic growth is relatively extensive, and there is also a problem of excessive demand for energy. For China, quality-oriented economic growth should be the core to avoid excessive growth in energy demand.

(4) The most important measure to achieve the goal of carbon neutrality is to increase the share of renewable energy and work towards electrification of transportation. However, the randomness and volatility of renewable energy would pose a huge challenge to the stable operation of the power system. For a city like Beijing that relies on energy outside the region, its own energy acceptance also needs to be strengthened, including strengthening energy storage construction, actively researching and promoting carbon capture and utilization of gas-fired units, which are effective ways to achieve carbon neutrality target. Simultaneously transportation electrification should focus on the development of two-way charging. In this way, excessive pressure on the power system could be avoided and the role of distributed energy storage can be achieved.
